# Flexibly tunable high-quality-factor induced transparency in plasmonic systems

**DOI:** 10.1038/s41598-018-19869-y

**Published:** 2018-01-24

**Authors:** Hua Lu, Xuetao Gan, Dong Mao, Baohua Jia, Jianlin Zhao

**Affiliations:** 1 MOE Key Laboratory of Material Physics and Chemistry under Extraordinary Conditions, and Shaanxi Key Laboratory of Optical Information Technology, School of Science, Northwestern Polytechnical University, Xi’an, 710072 China; 2 Centre for Micro-Photonics, Faculty of Science, Engineering and Technology, Swinburne University of Technology, Hawthorn, Victoria, 3122 Australia

## Abstract

The quality (Q) factor and tunability of electromagnetically induced transparency (EIT)-like effect in plasmonic systems are restrained by the intrinsic loss and weak adjustability of metals, limiting the performance of the devices including optical sensor and storage. Exploring new schemes to realize the high Q-factor and tunable EIT-like effect is particularly significant in plasmonic systems. Here, we present an ultrahigh Q-factor and flexibly tunable EIT-like response in a novel plasmonic system. The results illustrate that the induced transparency distinctly appears when surface plasmon polaritons excited on the metal satisfy the wavevector matching condition with the guided mode in the high-refractive index (HRI) layer. The Q factor of the EIT-like spectrum can exceed 2000, which is remarkable compared to that of other plasmonic systems such as plasmonic metamaterials and waveguides. The position and lineshape of EIT-like spectrum are strongly dependent on the geometrical parameters. An EIT pair is generated in the splitting absorption spectra, which can be easily controlled by adjusting the incident angle of light. Especially, we achieve the dynamical tunability of EIT-like spectrum by changing the Fermi level of graphene inserted in the system. Our results will open a new avenue toward the plasmonic sensing, spectral shaping and switching.

## Introduction

Electromagnetically induced transparency (EIT) is a quantum destructive interference effect that occurs in atomic systems, inducing a narrow transparency window in the original absorption spectrum^1^. In the transparency window, the dispersion features of the atomic medium can be drastically modified, which has promising applications in nonlinear optics, optical data storage and ultrafast optical switching^2^. Until now, the EIT effect has been demonstrated in various media. Behroozi *et al*. reported the generation of an EIT in the ultracold gas of sodium atoms, and reduced the light speed to 17 m/s by means of the EIT effect^3^. Qi and Lazoudis *et al*. realized the EIT effect in the Doppler-broadened lithium gas^4^ and acetylene photonic mirocell^5^. The EIT phenomenon was also observed by Lazoudis *et al*. in the sodium molecules generated within the heat-pipe oven^6^. In the classical systems, recently, a new kind of optical phenomena was observed and regarded as the EIT-like effect due to its remarkable analog of the EIT effect in atomic systems^7–10^. This kind of EIT-like effect attracts wide attentions because of its dispersion properties similar to those of EIT in atomic systems^7–10^. Especially, the EIT-like effect could find significant applications in chip-scale optical signal processing^8^. Naweed *et al*. observed the EIT-like response in coupled fused-silica microspheres and found the mode interference mechanism of EIT-like effect^7^. Xu *et al*. experimentally demonstrated the formation of EIT-like spectrum in all-optical silicon resonator systems^8^. Xiao *et al*. theoretically proposed the all-optical generation of multiple EIT in the drop-filter waveguide systems^9^. The performance (e.g., footprint and light confinement) of the traditional optical systems are inevitably restrained by the diffraction limit of light. Surface plasmon polaritons (SPPs) enable to confine light at sub-wavelength scale and overcome the diffraction limit of light, providing an excellent platform for nanoscale plasmonic control and elements^11–28^. Fortunately, the analog of the EIT effect was successfully observed in plasmonic systems such as plasmonic metamaterials^11–17^ and waveguides^18–21,28^. The EIT-like response in plasmonic systems could find the particular applications in ultrasmall photonic devices, for example the slow-light elements^20^, nanoscopic coherent light source^21^, nanosensors^27^ and nanofilters^28^. The high quality (Q) factor and dynamic tunability of plasmonic EIT-like response are of importance for improving the device performance and broadening the applications of EIT-like effect^27–31^. However, the Q factor and tunability of plasmonic EIT-like effect are generally hindered by the intrinsic loss and weak controllability of metal-based plasmonics in visible and near-infrared regions^11,20^. Exploring new ways to realize flexibly tunable high-Q-factor EIT-like effect in plasmonic systems is particularly meaningful and challenging.

Here, we propose a novel plasmonic system composed of a dielectric grating and a metallic film coated on the dielectric layers, and investigate its absorption spectral characteristics. The results illustrate that a classical analog of EIT effect can be observed in the absorption spectrum due to the satisfaction of wavevector matching condition between the SPP mode on the metallic film and guided mode in the high-refractive index (HRI) layer. The excited SPP mode destructively interferes with the coupled guided mode, resulting in the vanishment of SPP field and the appearance of transparency window in the absorption spectrum. Especially, we find that the EIT-like spectrum possesses an ultrahigh Q factor of >2000 and can be significantly tailored by adjusting the grating width, spacer thickness, HRI layer thickness and refractive index of HRI layer. By tuning the incident angle of light, a controllable EIT pair can be generated in the splitting absorption spectrum. Moreover, the flexible tunability of EIT-like spectrum is achieved by adjusting the Fermi level of graphene inserted in the HRI layer. The results may provide a new pathway toward the high-efficiency plasmonic sensing, spectral shaping and switching.

## Results

### Model and analytical theory for SPPs

**Figure 1:**
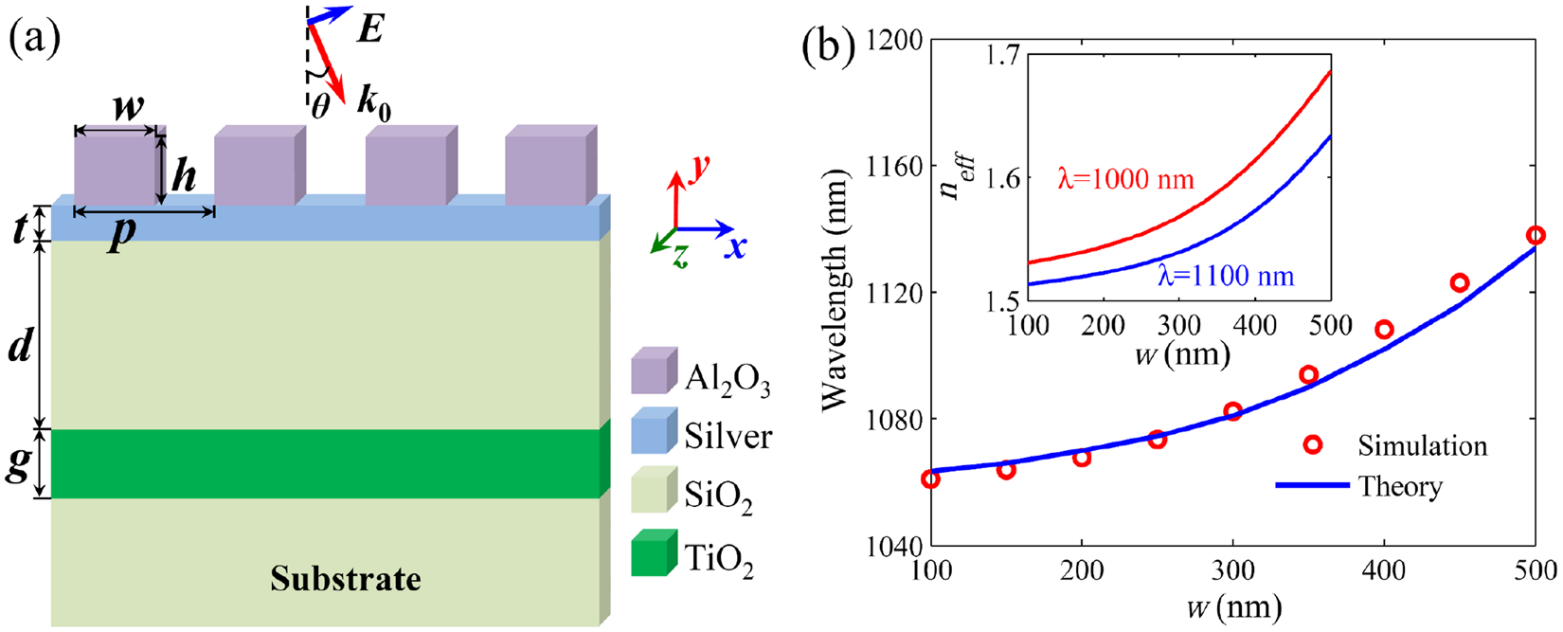
Schematic and excited SPP wavelengths. (**a**) Schematic of the plasmonic system composed of a Al_2_O_3_ (*n*_*a*_ = 1.76) grating and a silver film coated on dielectric layers. *p*, *w* and *h* are the pitch, width and height of Al_2_O_3_ grating, respectively. *t*, *g* and *d* are the thicknesses of silver, HRI (TiO_2_, *n*_*t*_ = 2.13) and spacer (SiO_2_, *n*_*s*_ = 1.45) layers, respectively. *θ* is the incident angle of light. (**b**) Wavelengths of excited SPP modes as a function of *w*. The inset shows the effective refractive indices (ERI) of SPP modes with different *w*.

As shown in Fig. 1(a), the plasmonic system consists of a Al_2_O_3_ grating and a metallic (silver) film coated on the SiO_2_/TiO_2_/SiO_2_ layers. In this system, *p*, *w* and *h* stand for the pitch, width and height of the Al_2_O_3_ grating, respectively. *t* and *g* represent the thicknesses of metal and TiO_2_ layers, respectively. *d* is the thickness of SiO_2_ spacer between the metal and TiO_2_ layers. Firstly, the light is assumed to be normally incident (*θ* = 0). The dielectric grating is used to compensate the wavevector mismatch between the incident light and SPPs for the excitation of transverse magnetic (TM) SPP mode on the metallic film. It should be noted that the function of this grating is different from that of surface grating structure in diode lasers^32^. For the multilayer with a metallic film, the dispersion relation of the SPP mode can be derived from the Maxwell’s equations and the boundary conditions. The SPP dispersion relation can be described as

1 $${{\rm{e}}}^{-2{k}_{a}h}=\frac{(1+{\varepsilon }_{a}{k}_{0})}{(1+{\varepsilon }_{c}{k}_{a})}\frac{(1+\psi )(1+\phi )+(1-\psi )(1-\phi ){{\rm{e}}}^{-2{k}_{m}t}}{(1-\psi )(1+\phi )+(1+\psi )(1-\phi ){{\rm{e}}}^{-2{k}_{m}t}}.$$

In Eq. (1), *ψ* = *ε*_*a*_*k*_*m*_/*ε*_*m*_*k*_*a*_ and *φ* = *ε*_*m*_*k*_*s*_/*ε*_*s*_*k*_*m*_. Here, *k*_*a*_ = (*β*^2^_*SPP*_ − *ε*_*a*_*k*_0_^2^)^1/2^, *k*_*m*_ = (*β*^2^_*SPP*_ − *ε*_*m*_*k*_0_^2^)^1/2^ and *k*_*s*_ = (*β*^2^_*SPP*_ − *ε*_*s*_*k*_0_^2^)^1/2^ are the wavevectors of light in the Al_2_O_3_ grating, metal and SiO_2_ layers, respectively. *β*_*SPP*_ = *k*_0_*n*_*eff*_ is the SPP propagation constant and *n*_*eff*_ is the effective refractive index (ERI) of SPP mode. *k*_0_ = 2π/*λ* is the wavevector of incident light, and *λ* is the incident wavelength. *ε*_*a*_ = *ε*_*c*_(1 − *f*) + *ε*_*A*_ *f* is equivalent to the relative permittivity of grating layer, and *f* = *w*/*p* is the duty cycle of grating^33^. *ε*_*m*_, *ε*_*s*_ (=*n*_*s*_^2^), *ε*_*A*_ (=*n*_*a*_^2^) and *ε*_*c*_ (=1) are the relative permittivities of the metal, SiO_2_, Al_2_O_3_ and air, respectively. The relative permittivity of the metal can be described by the Drude model: *ε*_*m*_ = *ε*_∞_ − *ω*_*p*_^2^/[*ω*(*iγ* + *ω*)]^34^, where *ω* = 2πc/*λ* is the angular frequency of light, and c is the speed of light in vacuum. *ε*_∞_, *γ* and *ω*_*p*_ stand for the relative permittivity at the infinite frequency, electron collision frequency and bulk plasma frequency, respectively. For silver, these parameters can be set as *ε*_∞_ = 3.7, *γ* = 0.018 eV and *ω*_*p*_ = 9.1 eV^35^. The SPP mode on the metallic film can be effectively excited once the following phase-matching condition is satisfied,

2 $${\rm{Re}}({\beta }_{SPP})-{k}_{0}\,\sin \,\theta =\pm m\frac{2{\rm{\pi }}}{p},$$

where the fundamental mode (i.e., *m* = 1) is considered in the wavelength range of interest. By combining Eqs (1) and (2), we can achieve the theoretical wavelengths of SPP modes. As depicted in Fig. 1(b), the SPP wavelength has a red shift with increasing Al_2_O_3_ grating width *w*. There exist the symmetric and antisymmetric SPP modes in silver films^36^. The SPP mode is antisymmetric in our structures (see Supplementary Information).

**Figure 2:**
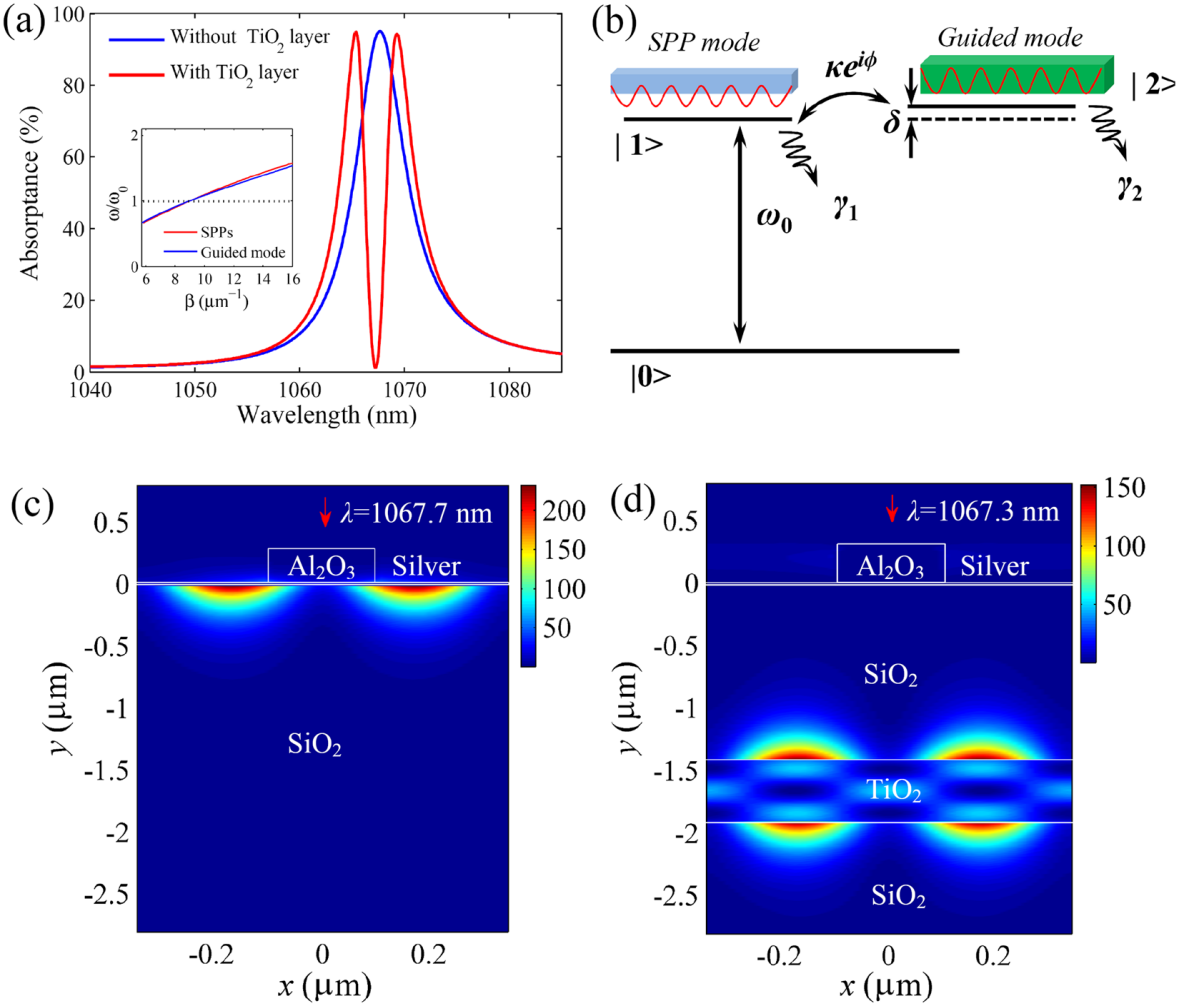
Spectral response, theoretical model and field distributions of EIT-like effect. (**a**) Absorption spectra of the structure without and with the TiO_2_ layer. The inset shows the dispersion relations of the SPP mode on the silver film and the guided mode in the TiO_2_ layer. (**b**) Coupled three-level model of the EIT-like effect in our system. (**c**) Field distribution |*E*|^2^ at the absorption wavelength (*λ* = 1067.7 nm) in the structure without the TiO_2_ layer (see Supplementary Video 1). (**d**) Field distribution |*E*|^2^ at the transparency wavelength (*λ* = 1067.3 nm) in the structure with the TiO_2_ layer (see Supplementary Video 2). The red arrows denote the direction of incident light. Here, *h* = 250 nm, *w* = 200 nm, *t* = 20 nm, *p* = 700 nm, *d* = 1400 nm and *g* = 495 nm.

To verify the theoretical results, we utilize the finite-difference time-domain (FDTD) method to simulate the optical response in the system. In the FDTD simulations, the perfectly matched layer absorbing boundary conditions are set at the top and bottom of computational space, and the periodic boundary conditions are set on the right and left sides of unit cell^37^. The real-world imperfections are not considered in the simulations, which may induce the deviation of about 5% for the spectral height between the simulations and experiments^8^. The proposed structures can be confidently realized by standard film deposition and nanofabrication equipment. As shown in Fig. 2(a,c), the structure exhibits a strong light absorption at the SPP wavelength due to the excitation of SPP mode (see Supplementary Video 1). It can be seen in Fig. 1(b) that the numerical simulations agree well with the theoretical results. To explain the shift of SPP wavelength with changing *w*, we plot the ERI (*n*_*eff*_) of SPP mode as a function of *w* by solving Eq. (1). The inset of Fig. 1(b) shows that *n*_*eff*_ ascends with increasing *w*. From Eq. (2), we can see that *β*_*SPP*_ is independent on *w*. Thus, it is reasonable to observe the red shift of SPP wavelength with the increase of *w*.

### Guided-mode resonance and generation of EIT-like effect

When a HRI (TiO_2_) film is sandwiched in the SiO_2_ layer, the guided-mode resonance can be established in the HRI layer. The dispersion relation of the TM guided mode is governed by

3 $${\kappa }_{g}g=n{\rm{\pi }}+2\,{\rm{arc}}\,\tan (\frac{{\varepsilon }_{s}}{{\varepsilon }_{t}}\frac{{K}_{s}}{{\kappa }_{g}}),$$

where *κ*_*g*_ = *(ε*_*t*_*k*_0_^2^ − *β*^2^)^1/2^ and *K*_*s*_ = (*β*^2^ − *ε*_*s*_*k*_0_^2^)^1/2^ are the wavevectors of propagation modes in the TiO_2_ and SiO_2_ layers, respectively. *ε*_*t*_ (=*n*_*t*_^2^) is the relative permittivity of TiO_2_ layer. Here, the guided mode order *n* is set as 1. When the incident light only passes through the dielectric layers, the guided mode in HRI layer can not be excited directly (see Supplementary Information). As depicted in Fig. 2(a), a narrow transparency window occurs in the middle of the original absorption spectrum. By solving Eqs (1) and (3), the obtained dispersion curves of SPP and guided modes intersect at the transparency wavelength, as shown in the inset of Fig. 2(a). The SPP and guided modes will strongly couple with each other through the evanescent fields under the wavevector matching condition. The coupling between plasmonic and photonic modes contributes to the formation of EIT-like spectrum^38^. This is different from the coupling between localized and delocalized SPPs in graphene EIT-like systems^39^. The low-loss dielectric structure can effectively improve the Q factor of EIT-like effect^8^. To further analyze the mechanism of the EIT-like effect, we plot a prototype three-level system in Fig. 2(b). Here, the SPP mode on the metal is in analogy to the upper state |1〉. When the light (analogous to the probe field) is incident on the structure, the SPP mode will be excited on the metal (corresponds to the transition from the ground state |0〉 to |1〉). When the wavevector of SPP mode approaches that of guided mode (in analogy to the state |2〉), the strong coupling (analogous to the pump field) will be generated through the evanescent fields between the SPP and guided modes (corresponds to the transition between |1〉 and |2〉). The guided mode in HRI layer can not be directly excited, which means that the transition from |0〉 to |2〉 is forbidden. Thus, the two possible pathways: |0〉 → |1〉 and |0〉 → |1〉 → |2〉 → |1〉 will destructively interfere and generate the EIT-like effect^12,40^. As shown in Fig. 2(d), the destructive interference between the SPP and guided modes results in the disappearance of SPP field (see Supplementary Video 2), which reduces the SPP loss and gives rises to the high Q-factor EIT-like spectral response.

### Analytical theory for EIT-like effect

**Figure 3:**
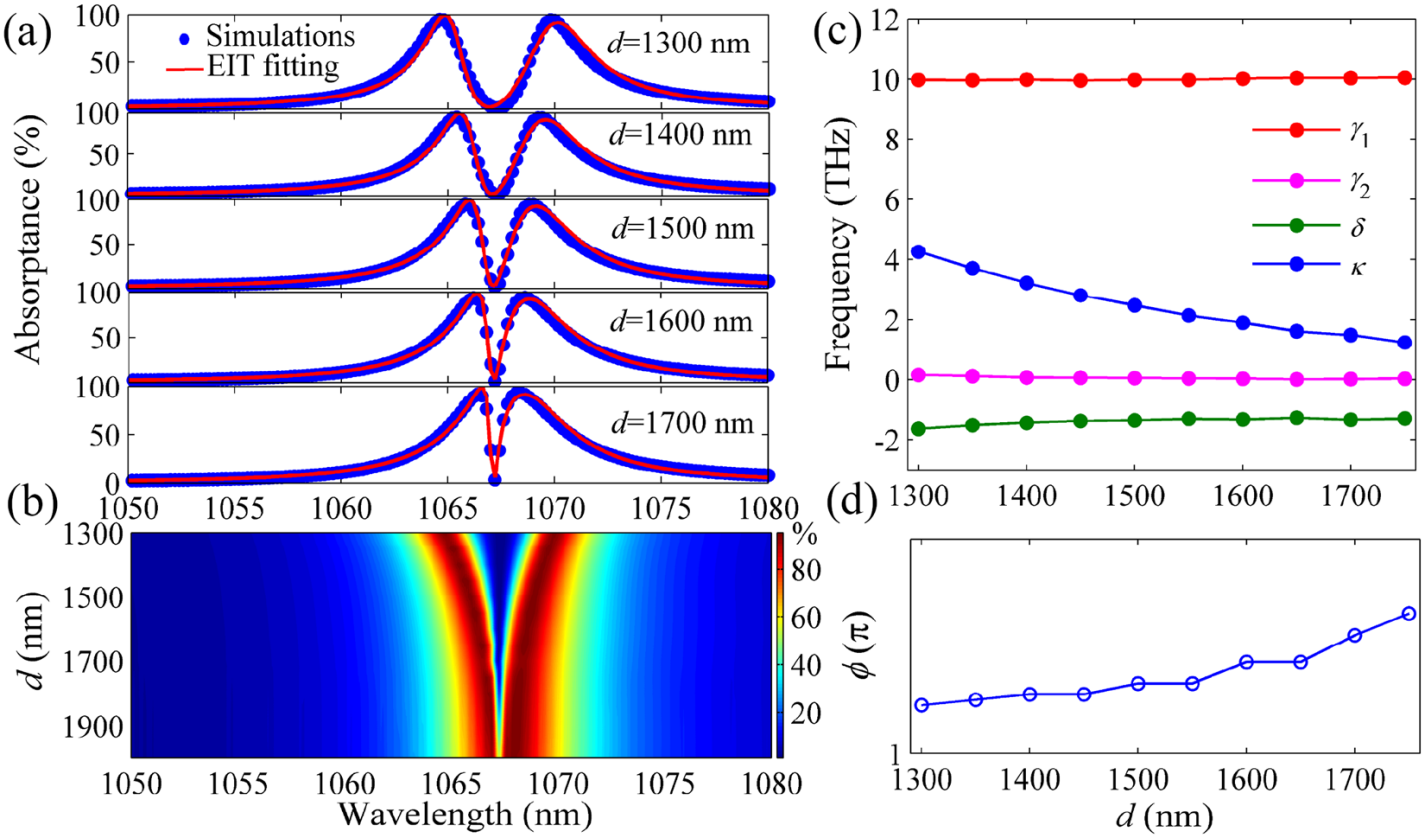
EIT-like response dependent on the thickness of spacer layer and fitting values of the parameters in EIT model. (**a**) Absorption spectra with different thicknesses of spacer layer *d*. The dots and curves denote the FDTD simulation and theoretical fitting results, respectively. (**b**) Evolution of absorption spectra with *d*. (**c**) Fitting values of the parameters *γ*_1_, *γ*_2_, *κ* and *δ* with different *d*. (**d**) Fitting results of *ϕ* as a function of *d*. Here, *h* = 250 nm, *w* = 200 nm, *t* = 20 nm, *p* = 700 nm and *g* = 495 nm.

The two-oscillator EIT model enables to quantitatively describe the EIT-like response^12^. As shown in Fig. 1, the light is incident on the metallic film with the Al_2_O_3_ grating and excites the SPP mode (i.e., oscillator 1). The guided mode in the TiO_2_ layer (i.e., oscillator 2) is established only by coupling with oscillator 1. As depicted in Fig. 2(b), *ω*_0_ stands for the resonance frequency of oscillator 1 (SPP frequency), *δ* is the resonance frequency detuning between oscillators 1 and 2, *γ*_1_ and *γ*_2_ are the decaying rates from the loss in oscillators 1 and 2, respectively. *κe*^*iϕ*^ is the coefficient of coupling between oscillators 1 and 2, and *ϕ* is the coupling phase retardation^40^. When *γ*_2_ ≪ *γ*_1_ ≪ *ω*_0_, |*δ*| ≪ *γ*_1_ and |*ω*−*ω*_0_| ≪ *ω*_0_, the light absorptance of the entire system can be expressed as^40^

4 $$A={\rm{Im}}\{\frac{F(\omega -{\omega }_{0}+\delta +i{\gamma }_{2}/2)}{{(\kappa {e}^{i\varphi })}^{2}-(\omega -{\omega }_{0}+i{\gamma }_{1}/2)(\omega -{\omega }_{0}+\delta +i{\gamma }_{2}/2)}\},$$

where *A* is the imaginary part of the result obtained by solving the coupled differential equations (see Methods section). *A* means the ratio of the power of absorption light in the system to the power of incident light. *F* is an amplitude coefficient. According to Eq. (4), we can fit the simulation results of absorption spectra. As shown in Fig. 3(a), the fitting curves agree well with the simulation results, which verifies the reasonability of theoretical model. From Fig. 3(b), we can see that the EIT-like spectral width becomes narrower at the same wavelength when *d* increases, giving rise to the higher Q-factor induced transparency. The absorption dip increases with *d*, and the spectral widths on both sides of the EIT window nearly keep constant. When *d* increases from 1300 to 1700 nm, the dip value of EIT-like spectrum changes slowly due to the relatively strong coupling between the SPP and guided modes. When *d* further increases, the coupling gradually becomes weak, and thus the metal-based plasmonic loss results in the shallower absorption dip, as shown in Fig. 3(b). The Q factor of EIT-like spectrum can exceed 2000 when *d* > 1700 nm. It is not superior to the Q factor in all-dielectric structures^8^, but is one order of magnitude larger than that of other plasmonic systems such as plasmonic metamaterials^11^ and waveguides^18–20^. If the multiple TiO_2_ layers are introduced in the system, the absorption spectrum is further split, forming higher Q-factor multiple EIT windows (see Supplementary Information). By fitting the simulation results, we can obtain the theoretical values of physical parameters in the EIT model. We plot the physical parameters *γ*_1_, *γ*_2_, *κ*, *δ*, and *ϕ* as a function of *d*, as shown in Fig. 3(b,c). It is found that the coupling strength *κ* between the SPP and guided modes successively decreases with increasing *d*. The detuning *δ* is about −1.34 THz when *d* approaches 1700 nm, and the decay rate *γ*_1_ is 10 THz, which is ~500 times larger than the decay rate *γ*_2_ (*γ*_2_ = 0.02 THz). The phase retardation *ϕ* ascends slowly with increasing *d*, as shown in Fig. 3(c). Here, *γ*_1_ and *γ*_2_ can be respectively regarded as the dephasing rates of the SPP and guided modes, which nearly keep constant with the change of *d*. *κ* corresponds to the Rabi frequency in the EIT system^41^. The width of the EIT-like spectrum becomes sharper with the decrease of the Rabi frequency. For the light passing through the system, the slow-light effect will be generated due to the strong dispersion in the transparency window (see Supplementary Information). The group index can be derived from the above theoretical model^11^. We find that the group index exceeds 800 in the plasmonic system with *d* = 1400 nm, which is one order of magnitude higher than that of plasmonic metamaterials^11^ and waveguides^20^.

### Dependence of EIT-like spectrum on physical parameters

**Figure 4:**
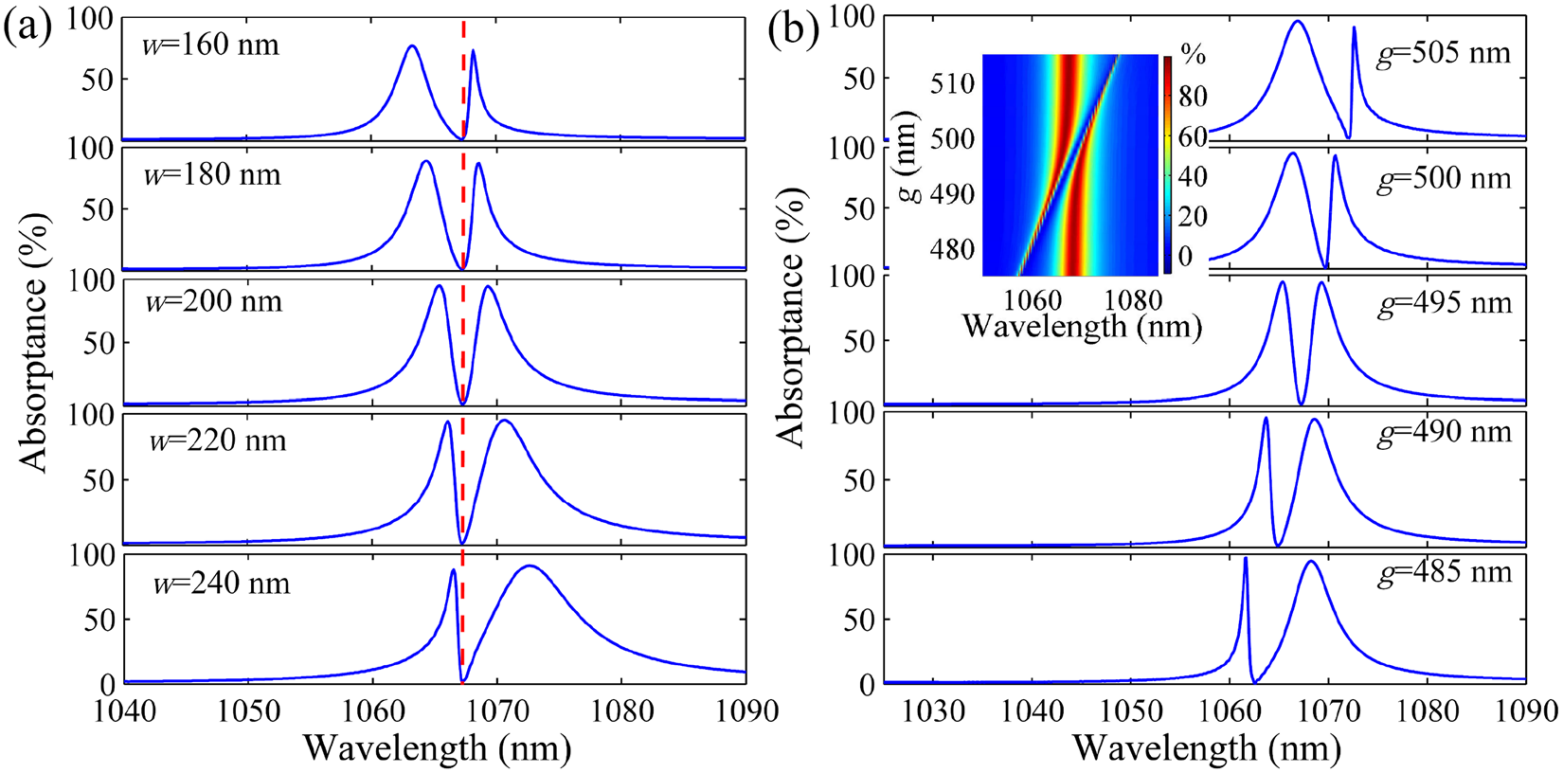
EIT-like response dependent on the grating width and thickness of HRI layer. Absorption spectra of the structure (**a**) with different *w* when *g* = 495 nm and (**b**) with different *g* when *w* = 200 nm. The inset shows the evolution of absorption spectrum with *g*. Here, *h* = 250 nm, *t* = 20 nm, *p* = 700 nm and *d* = 1400 nm.

**Figure 5:**
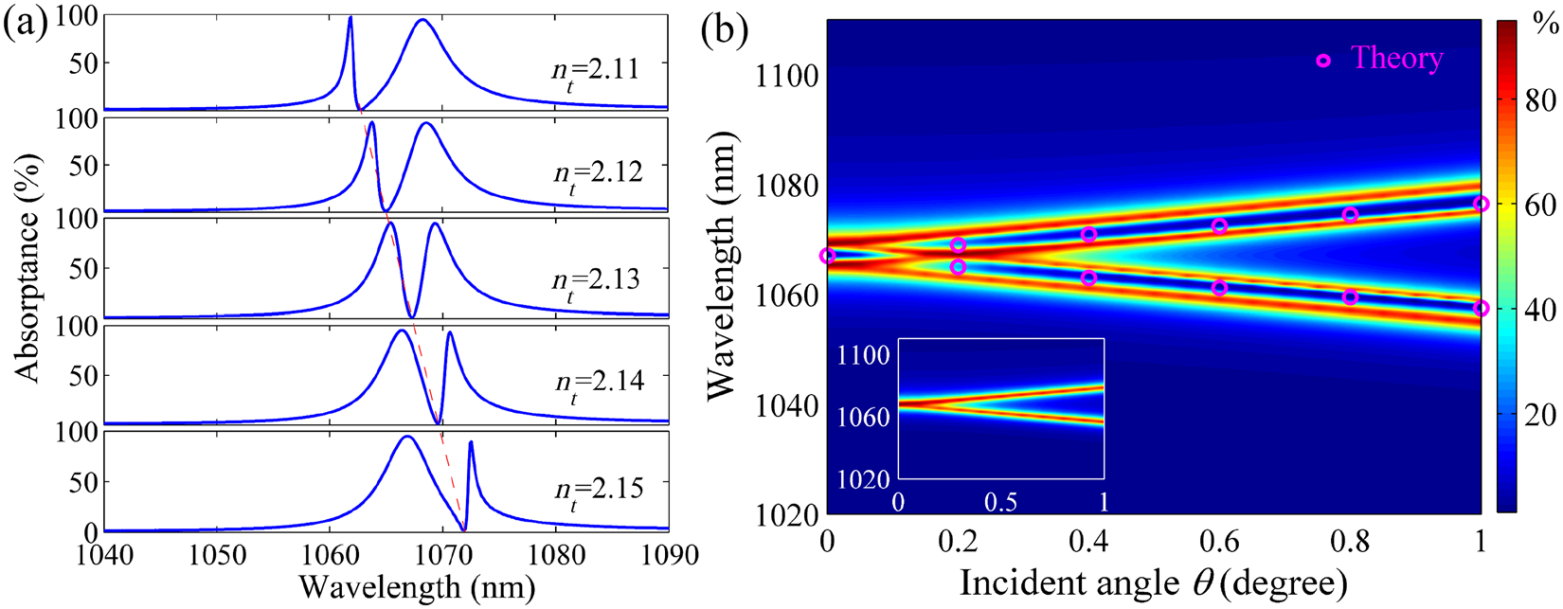
EIT-like response dependent on the refractive index of HRI layer and incident angle of light. (**a**) Absorption spectra of the structure with different refractive indices (*n*_*t*_) of the HRI layer. (**b**) Evolution of absorption spectrum with *θ* in the structure with the TiO_2_ layer. The circles present the theoretical wavelengths of guided mode in the TiO_2_ layer. The inset shows the absorption spectrum evolution with *θ* in the structure without the TiO_2_ layer. Here, *h* = 250 nm, *w* = 200 nm, *t* = 20 nm, *p* = 700 nm, *d* = 1400 nm and *g* = 495 nm.

We investigate the dependence of the EIT-like spectrum on the Al_2_O_3_ grating width *w*, which controls the wavelength of SPP mode. As shown in Fig. 4(a), the EIT-like spectral profile is sensitive to *w*, but the position of transparency window nearly maintains unchanged. As mentioned above, the SPP wavelength exhibits a red shift with increasing *w*, while the wavelength of guided mode is fixed because of the phase matching condition. Thus, the spectrum becomes asymmetric and steep on the right (left) side of induced transparency when *w* is smaller (larger) than 200 nm owing to the deviation between the SPP and guided-mode wavelengths. Moreover, we study the influence of the TiO_2_ layer thickness *g* on the EIT-like spectrum. As shown in Fig. 4(b), the wavelength of induced transparency possesses a red shift when *g* increases, which results in the asymmetric spectra. This behavior can also be explained by the wavevector matching condition. The ERI of the guided mode in the TiO_2_ layer increases with *g* (see Supplementary Information), thus the guided-mode wavelength inevitably raises to match the wavevector of the SPP mode. The results could find applications in the spectral shaping and optical filtering. In the inset of Fig. 4(b), we can see the obvious EIT-like spectra when *g* is altered from 480 to 510 nm. It is found that the EIT wavelength has a red shift as the refractive index of HRI layer increases, as shown in Fig. 5(a). If the HRI layer is employed as a channel to pass through transparent fluidic media, the plasmonic system can work as a refractive index sensor by detecting the reflection of incident light. The figure of merit (FOM) of the sensor is about 80, which is one order of magnitude larger than that of the sensors based on the EIT-like effect in plasmonic metamaterials^27^. When a denser HRI layer (e.g. Si_3_N_4_) is employed, the obvious EIT-like spectrum can be achieved with selecting a larger *g* (see Supplementary Information). In addition, the EIT-like response can be obviously generated at the same wavelength when the dissipative loss of metal changes (see Supplementary Information). These features contribute to the flexible selection of HRI and metal materials in the experiments.

From Eq. (2), we can see that the SPP mode is dependent on the incident angle of light *θ*, which thereby enables to realize the angle-tuned EIT-like spectrum. Figure 5(b) depicts the evolution of absorption spectrum with *θ*. It shows that an obvious EIT pair (i.e., double EIT-like transparency windows) is formed in the splitting absorption spectrum with increasing *θ*. This phenomenon can be interpreted by the wavevector matching mechanism. When the light obliquely impinges on the system, two different SPP modes in the ± *x* axis directions will be generated on the metal, corresponding to the symbol ±*m* (*m* = 1) in Eq. (2). The propagation constant of the SPP mode will increase (decrease) for +1 (−1) order mode with increasing *θ*, which gives rise to the blue (red) shift for SPP wavelengths. It results in the splitting of absorption spectrum, as shown in the inset of Fig. 5(b). To remain the match between the wavevectors of the SPP and guided modes, meanwhile, the guided-mode wavelength will possess a blue (red) shift for +1 (−1) order SPP mode. Thus, the coupling of guided modes destructively interferes with the SPP modes, giving rise to the generation of the EIT pair. By solving Eqs (2) and (3), we can obtain the theoretical wavelengths of guided modes, which are in accordance with the positions of transparency windows of the EIT pair, as shown in Fig. 5(b). The theoretical results agree well with the FDTD simulations. These results will offer a significant guide for the geometrical design in experiments. The angle sensitivity is ~10 nm/degree, which is higher than the reports in other metallic structures^42,43^. In practice, the collimation package can be used to reduce the influence of light beam divergence on the EIT-like response.

### Tunability of EIT-like response based on grapheme

**Figure 6:**
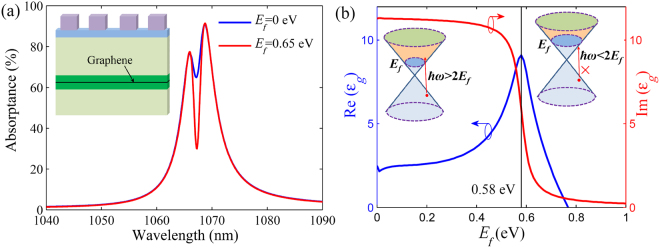
Graphene-controlled EIT-like response. (**a**) Absorption spectra of the graphene-assisted structure with different Fermi levels *E*_*f*_ for graphene. The inset shows the graphene-assisted structure. (**b**) Real and imaginary parts of the relative permittivities of graphene with different *E*_*f*_ at the wavelength of 1067.3 nm. The insets describe the interband transitions of graphene with different *E*_*f*_. Here, *h* = 250 nm, *w* = 200 nm, *t* = 20 nm, *p* = 700 nm, *d* = 1700 nm and *g* = 495 nm.

Finally, we investigate the active control of the EIT-like response, which is crucial for the realization of active photonic devices. Graphene, a two-dimensional (2D) crystal of carbon atoms, attracts broad attentions because of its excellent properties containing the ultra-wide operating wavelength range and ultra-high carrier mobility^44–47^. Especially, the surface conductivity of graphene relies on the Fermi level *E*_*f*_, which can be dynamically tuned via chemical doping or gate voltage^46–49^. The graphene can facilitate the active modulation of light in photonic structures^48,49^. We propose to insert a graphene monolayer in the middle of the HRI layer for the sufficient interaction between the guided mode and graphene, which is achievable in the experiments^50^. Due to the interband transition of electrons in graphene, the photons of energy *ħω* > 2*E*_*f*_ for the guided mode will be absorbed by graphene, which hinders the generation of EIT-like spectrum. This mechanism is different from that of graphene plasmonic systems^51^. Here, the surface conductivity *σ*_*g*_ of graphene can be derived according to the random-phase approximation in the local limit (see Methods section). The carrier mobility of graphene is assumed as 10000 cm^2^V^−1^s^−1^. Thus, the relative permittivity of graphene can be set as *ε*_*g*_ = 2.5 + *iσ*_*g*_/(*ωε*_0_Δ), where Δ = 0.34 nm is the practical thickness of graphene monolayer. As shown in Fig. 6(a), the EIT-like spectrum becomes not obvious when *E*_*f*_ = 0 eV, while a narrow transparency window distinctly appears when *E*_*f*_ = 0.65 eV at the wavelength of 1067.3 nm. This is because the photon energy at this wavelength is ~1.16 eV less than 2*E*_*f*_ (=1.3 eV), and thus the incident light can not be strongly absorbed by graphene monolayer. As shown in Fig. 6(b), the imaginary part of graphene relative permittivity corresponds to the dissipative loss of graphene, which drastically decreases with increasing *E*_*f*_ near 0.58 eV at the wavelength of 1067.3 nm. Thus, the light absorption of the system will descend when *E*_*f*_ increases. By fitting the spectra with the theoretical model, we find that *γ*_2_ decreases from 1.88 THz to 0.74 THz when *E*_*f*_ changes from 0 eV to 0.65 eV, while *γ*_1_ is almost unchanged. Therefore, the EIT-like spectrum is dependent on the dephasing rate of guided mode, which can be controlled by the Fermi level of graphene. When the graphene is placed above the HRI layer, the EIT-like spectrum can also be tuned by adjusting *E*_*f*_. It is difficult for the ultrathin graphene to affect the wavelength of guided mode in HRI layer, so the induced transparency position is not sensitive to the change of the Fermi level. If the HRI layer is a stack of 2D media (e.g. graphene and MoS_2_)^52,53^, the tunable EIT-like response can also be achieved (see Supplementary Information). The graphene-controlled EIT-like spectrum provides a promising avenue to realize active optical devices such as switches and modulators^46^.

## Discussion

We have presented a flexibly tunable and ultrahigh Q-factor EIT-like effect in a new plasmonic system composed of a Al_2_O_3_ grating and a silver film coated on the SiO_2_/TiO_2_/SiO_2_ layers. The results show that the induced transparency is generated in the strong absorption spectrum when the SPP mode excited on the silver film satisfies the wavevector matching condition with the guided mode in the (TiO_2_) HRI layer. The Q factor of EIT-like response can exceed 2000, which is one order of magnitude larger than that of other plasmonic systems such as plasmonic metamaterials and waveguides. The lineshape and position of EIT-like spectrum can be tailored by controlling the spacer thickness, grating width, HRI layer thickness and refractive index of HRI layer. When the light is obliquely incident, an EIT pair is generated in the splitting absorption spectrum, which can be tuned by adjusting the incident angle of light. Particularly, we introduce graphene in the HRI layer and achieve the dynamic tunability of EIT-like response by controlling the Fermi level of graphene. These results could find significant applications in high-performance plasmonic sensing, spectral shaping and switching.

## Methods

To theoretically analyze the EIT-like effect, the simple two-oscillator EIT model can be utilized to quantitatively describe the spectral response^12,14,41^. In plasmonic systems, the formula of the light absorptance can be derived from the coupled differential equations, which are described as follows,

5 $$\{\begin{array}{c}\frac{{\partial }^{2}{q}_{1}(t)}{\partial {t}^{2}}+{\gamma }_{1}\frac{\partial {q}_{1}(t)}{\partial t}+{\omega }_{0}^{2}{q}_{1}(t)+{\kappa }_{c}{q}_{2}(t)=\eta {E}_{ex}(t)\\ \frac{{\partial }^{2}{q}_{2}(t)}{\partial {t}^{2}}+{\gamma }_{2}\frac{\partial {q}_{2}(t)}{\partial t}+{({\omega }_{0}-\delta )}^{2}{q}_{2}(t)+{\kappa }_{c}{q}_{1}(t)=0,\end{array}$$

where *q*_1_(*t*) and *q*_2_(*t*) represent the field amplitudes in oscillators 1 and 2, respectively. *E*_*ex*_(*t*) is the incident electric field. *κ*_*c*_ = *κe*^*iϕ*^ is the coupling coefficient between oscillators 1 and 2. *η* stands for the coupling strength between the incident light and oscillator 1.

The surface conductivity of graphene can be derived by the random-phase approximation (RPA) in the local limit^54^. The surface conductivity of single-layer graphene can be quantitatively described as

6 $$\begin{array}{rcl}{\sigma }_{g} & = & i\frac{2{e}^{2}{k}_{B}T}{\pi {{\hbar}}^{2}(\omega +i{\tau }^{-1})}\,\mathrm{ln}[2\,\cos \,{\rm{h}}(\frac{{E}_{f}}{2{k}_{B}T})]\\  &  & +\frac{{e}^{2}}{4{\hbar}}[\frac{1}{2}+\frac{1}{\pi }{\rm{arc}}\,\tan (\frac{{\hbar}\omega -2{E}_{f}}{2{k}_{B}T})-\frac{i}{2\pi }\,\mathrm{ln}\,\frac{{({\hbar}\omega +2{E}_{f})}^{2}}{{({\hbar}\omega -2{E}_{f})}^{2}+{(2{k}_{B}T)}^{2}}],\end{array}$$

where *e* is the electron charge, *T* is the temperature, *k*_*B*_ is the Boltzmann’s constant, *ħ* is the reduced Planck’s constant, *ω* is the angular frequency of incident light in vacuum, *E*_*f*_ is the Fermi level of graphene and *τ* stands for the charge carrier relaxation time. For graphene, *τ* is dependent on the carrier mobility *μ* and could be obtained by *τ* = *μE*_*f*_/(*ev*_f_^2^). The previous reports showed that *μ* of graphene on the SiO_2_ layer could approach 40000 cm^2^V^−1^s^−1^ at room temperature^55^. In order to ensure the credibility of results, a reasonable carrier mobility of 10000 cm^2^ V^−1^s^−1^ is selected in the calculations. The Fermi velocity *v*_f_ is 10^6^ m/s.

## Electronic supplementary material

Supplementary Information

Supplementary Video 1

Supplementary Video 2
