# Supplementary material for: Flexibly tunable high-quality-factor induced transparency in plasmonic systems
Source: Sci Rep. 2018 Jan 24;8:1558. doi: 10.1038/s41598-018-19869-y (PMC5784153; doi:10.1038/s41598-018-19869-y)
Supplement: Supplementary file 1 — Supplementary Information [file 41598_2018_19869_MOESM1_ESM.doc]

**Supplementary Information**

Flexibly tunable high-quality-factor induced transparency in plasmonic systems

**Hua Lu^1,*^, Xuetao Gan^1^, Dong Mao^1^, Baohua Jia^2^ and Jianlin Zhao^1,†^**

^1^ MOE Key Laboratory of Material Physics and Chemistry under Extraordinary Conditions, and Shaanxi Key Laboratory of Optical Information Technology, School of Science, Northwestern Polytechnical University, Xi’an 710072, China

^2^ Centre for Micro-Photonics, Faculty of Science, Engineering and Technology, Swinburne University of Technology, Hawthorn, Victoria 3122, Australia

***^*^*** [*hualu@*](mailto:luhua@opt.ac.cn)*nwpu.edu.cn*

**^†^** *jlzhao@nwpu.edu.cn*

**1. Spectral response of the TiO_2_ layer**

Figure S1. Transmission and reflection spectral response of the TiO_2_ layer sandwiched in the SiO_2_ layers.

**2. ERI of guided mode in the HRI layer with different *g***

Figure S2. Effective refractive indices (*n_eff_*) of guided modes in the HRI layer with different thicknesses *g* of HRI layer.

**3. EIT-like response with multiple HRI (TiO_2_) layers**

As shown in Fig. S3, the absorption spectrum will further split when the TiO_2_ layer number increases. The multiple EIT response will be formed in the spectra when multiple TiO_2_ layers are introduced into the system. The spectral width of EIT dip will become narrower. For example, the EIT spectral width of the system with triple TiO_2_ layers is about two times narrower than that of the system with one TiO_2_ layers. Thus, the Q factor of EIT response can be further improved when the multiple TiO_2_ layers are inserted in the configuration.

Figure S3. Absorption spectra of the plasmonic systems with different TiO_2_ layer numbers. Here, the spacer thickness is set as 1700 nm. The distance between the adjacent TiO_2_ layers is also fixed as 1700 nm.

**4. EIT-like response with a denser HRI layer**


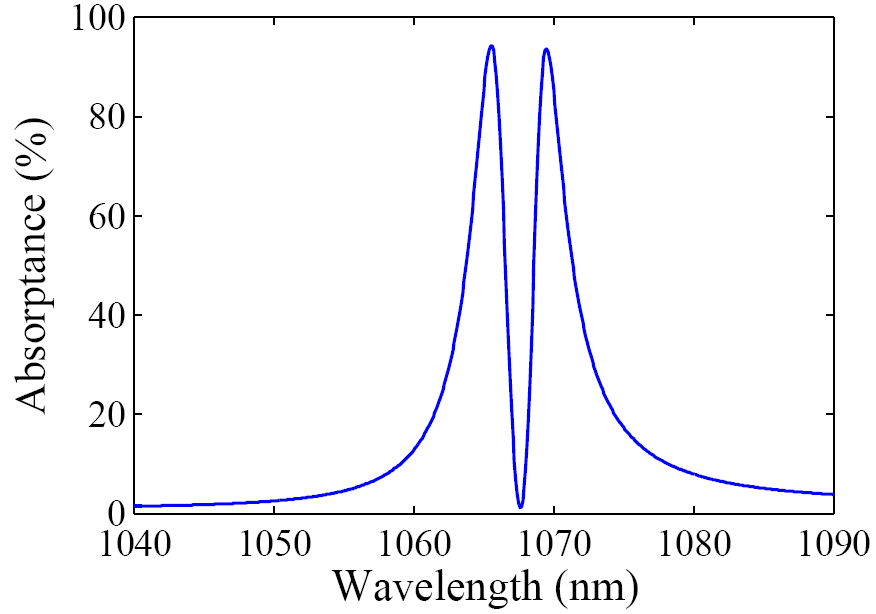


Figure S4. Absorption spectrum of the plasmonic system with the HRI (Si_3_N_4_, *n_t_*=2.2) layer when *g*=465 nm.

**5. Field distributions of SPP mode on the metallic film**

There exists two types of SPP modes on the sliver film, namely symmetric and antisymmetric modes ^S1^. We plot the electric and magnetic fields of the SPP mode. As shown in Fig. S5, we can see that the SPP mode on the metallic film is antisymmetric.

Figure S5. Electric and magnetic fields of SPP mode excited in the silver film.

**6. Controlled EIT with a stack of 2D media**

We calculate the spectral response in the structure when the HRI dielectric layer is constructed with a stack of 2D media (e.g. graphene and MoS_2_). The MoS_2_ layer is assumed as the dielectric host. The graphene layers are stacked among the host ^S2^. The simulation results in Fig. S6 show that the EIT response can also be generated in the system and controlled by adjusting the Fermi level of graphene.


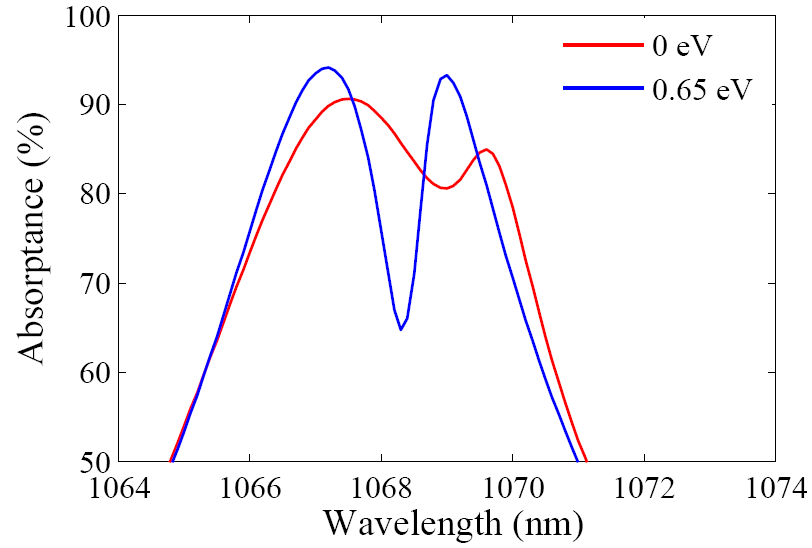


Figure S6. Absorption spectra in the system with a stacked 2D media when the graphene Fermi level *E_f_* is set as 0 and 0.65 eV.

**7. EIT-like response with different metal losses**

We consider the influence of metal loss on the EIT spectrum. The electron collision frequency (*γ*) in Drude model of metal stands for the intrinsic loss in metal ^S3^. We plot the EIT spectra in the system with different *γ* for the metal, as depicted in Fig. S7. It is shown that the EIT-like response can be obviously generated at the same wavelength with different metal losses.

Figure S7. EIT absorption spectra with different metal losses. Here, *h*=250 nm, *w*=200 nm, *t*=20 nm, *p*=700 nm, *d*=1700 nm, and *g*=495 nm.

**8. Slow-light effect in the plasmonic system**

The group index ( the group velocity) of transmitted light from the system can be estimated from the slope of the susceptibility *χ_r_* ^S4^, which can be derived from the two-oscillator model and depicted as

From Fig. S8, we can see that there is the strong dispersion in the transparency window. The group velocity is dependent on the wavelength and approaches the maximum value of 820 at the transparency peak.

(a)

(b)

Figure S8. (a) Susceptibility *χ_r_*. (b) Group index of light passing through the system. Here, *h*=250 nm, *w*=200 nm, *t*=20 nm, *p*=700 nm, *d*=1400 nm and *g*=495 nm.

**References**

S1. Andrew, P. & Barnes, W. Energy transfer across a metal film mediated by surface plasmon polaritons. *Science* **306**, 1002-1105 (2004).

S2. Mattheakis, M., Valagiannopoulos, C. A. & Kaxiras, E. Epsilon-near-zero behavior from plasmonic Dirac point: Theory and realization using two-dimensional materials. *Phys. Rev. B* **94**, 201404(R) (2016).

S3. Lu, H., Liu, X. & Mao, D. Plasmonic analog of electromagnetically induced transparency in multi-nanoresonator-coupled waveguide systems. *Phys. Rev. A* **85**, 053803 (2012).

S4. Zhang, S., Genov, D., Wang, Y., Liu, M. & Zhang, X. Plasmon-induced transparency in metamaterials. *Phys. Rev. Lett.* **101**, 047401 (2008).
